# Adding a Fibular Strut Allograft to Intramedullary Nail and Cancellous Autograft During Stage II of the Masquelet Technique for Segmental Femur Defects: A Technique Tip

**DOI:** 10.5435/JAAOSGlobal-D-19-00179

**Published:** 2020-07-07

**Authors:** Omar Ramos, Michael Mariorenzi, Joey P. Johnson, Roman A. Hayda

**Affiliations:** From the Department of Orthopaedic Surgery (Dr. Ramos and Dr. Johnson), Loma Linda University, Loma Linda, CA, and the Department of Orthopaedic Surgery (Dr. Mariorenzi and Dr. Hayda), Warren Alpert Medical School of Brown University, Providence, RI.

## Abstract

Reconstruction of segmental diaphyseal bone defects has been a major challenge in limb salvage surgery. Staged reconstruction as first described by Masquelet is a common strategy to deal with this problem in limb salvage surgery. One consequence of this technique is a time period of prolonged limited weightbearing while the segmental defect heals. The purpose of this study was to describe an adjunctive technique for stage II of the Masquelet procedure and retrospectively analyze the outcome and weight bearing progression of 3 patients who sustained femur fractures with significant bone loss and underwent this technique. A retrospective chart review was performed. The patients (2 males, 1 female with an average age of 36.6 years) all sustained segmental femur fractures which resulted in significant bone loss. Induced membrane technique with adjunct use of a fibular strut allograft was performed after initial stabilization and PMMA spacer placement. All three patients went on to union and full weight bearing after being treated by the described technique. All the patients were allowed toe-touch weight bearing immediately after surgery and all progressed to weight bearing as tolerated at an average of 3.6 months. Using a fibular strut allograft as an adjunct to the induced membrane technique serves as a biologic and mechanical scaffold and may allow earlier weightbearing.

Reconstruction of segmental diaphyseal bone defects is a major challenge in limb salvage surgery for both anatomic and functional reasons.^1^ The concept of membrane induction was first introduced by Masquelet et al. as a possible solution to complex reconstruction dilemmas.^2^ The principle of the induced membrane technique involves placing a cement spacer in the bone defect which provokes an inflammatory reaction to this foreign object with the formation of a vascularized pseudosynovial membrane.^3^ This method of reconstruction requires two stages. The first stage involves débridement and insertion of a polymethyl methacrylate (PMMA) cement spacer occupying the volume of the bone defect.^2^ The second stage involves removal of the PMMA spacer while maintaining the surrounding induced membrane and insertion of autograft cancellous bone and allograft bone graft when needed.^2^ Originally, this PMMA spacer was thought to be helpful as a structural support, but later studies demonstrated the important biological effects of the induced membrane that forms around the spacer because it is rich in growth factors such as transforming growth factor-beta, vascular endothelial growth factor, and bone morphogenetic protein-2.^3,4,5,6,7^ These growth factors are thought to be instrumental in the successful outcomes reported in the initial study by Masquelet and in the development of macroscopically normal bone noted in a postoperative cross sections.^4,6,8^

The PMMA cement spacer plays two roles. First, it temporarily occupies the space of the segmental defect, preventing fibrous tissue invasion of the recipient site. Second, it induces a peripheral membrane, a rich vascular bed for graft reimplantation, and prevents resorption of the graft.^1,3,6,8^ The PMMA spacer is also often used as an antibiotic delivery device to help minimize the risk of infection.

In recent years, the induced membrane technique has been expanded beyond lower extremity fractures and studies have been published touting its success in upper extremity bone loss injuries.^9,10,11,12,13^ The Masquelet technique has also been applied to pediatric disciplines, being described for pediatric trauma, reconstruction of pseudoarthroses, and reconstruction after resections of large tumors.^14,15,16,17,18,19,20,21,22,23^

One consequence of the Masquelet technique, as would be expected with segmental bone loss, is prolonged limited weight bearing on the affected extremity, following the second stage procedure.^2,24-26^ Our technique involves the use of a fibular strut allograft in addition to autograft during the second stage of the procedure that allows for earlier partial weight bearing and maintains the benefits of the induced membrane. We describe this technique with a case series of patients.

## Methods

After IRB approval, we retrospectively reviewed the clinical and radiographic documentation of three consecutive patients who sustained open, segmental femur fractures resulting in notable bone loss and were treated with the induced membrane technique described above by two of the authors between 2015 and 2019.

In stage one, after appropriate débridement, the surgical approach is dictated by the site of open injury. Adequate débridement is essential in the first stage of this technique. It is not uncommon that multiple débridements are necessary to obtain a clean, stable tissue bed. Once an adequate tissue bed is obtained, a PMMA spacer impregnated with antibiotics is placed in the segmental void.^2^

**Figure 1:**
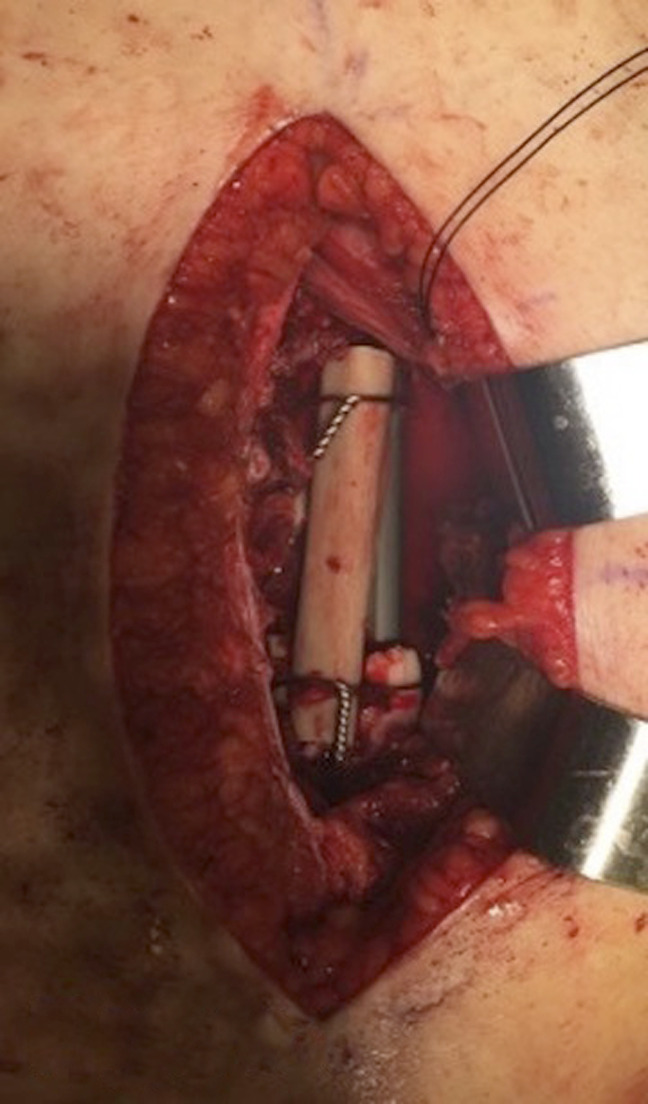
Case 2. Intraoperative photograph showing insets in femoral shaft with the fibular strut wired into place.

After the induced membrane has had an opportunity to mature, ideally for 4 to 6 weeks, the membrane is incised, and the PMMA spacer removed. The void is then packed with autograft bone and bone substitute as needed, as per the original Masquelet technique.^2^ In addition, a trough is created, either by the use of a sagittal saw or high speed burr, in the proximal femur fragment and distal femur fragment allowing for a fibular strut allograft to be inset into the fragments. The fresh-frozen allograft is cut to size with a sagittal saw and then inset into the femur. The inset with a flat “shelf” for the strut to mate to the proximal and distal ends is critical for it to participate in load sharing. It is not simply laid on. This allograft is then cerclage wired into place proximally and distally to prevent migration of the allograft (Figure 1). Conceptually, the strut allows for the load to be transmitted from the distal to the proximal bone segment during bone consolidation without complete reliance on the interlocking screws.

## Results

The patients ages ranged from 30 to 41. All patients were followed up until full union and full weight bearing without pain were achieved. The average follow-up was 20 months. All three patients achieved union and progressed to weight bearing as tolerated by an average of 3.6 months. Table 1 summarizes the weight-bearing progression after the second stage of the induced membrane technique, and the three cases are presented below.

**Table 1:** Case and Postoperative Weight-Bearing Progression After Second Stage Grafting Procedure

Patient	Weight Bearing
Case 1: 38-year-old male pedestrian hit by bus.	Immediate postop: WBAT for transfers, TTWB otherwise. 4 weeks post-op: Partial weight bearing (50% body weight) 8 weeks post-op: WBAT
Case 2: 40-year-old-female pedestrian hit by bus.	Immediate post-op: TTWB 3 months post-op: WBAT
Case 3: 30-year-old male scooter rider hit by vehicle.	Immediate post-op: TTWB 6 weeks post-op: partial weight bearing (25% -50% body weight) 10 weeks post-op: partial weight bearing (50%-75% body weight) 20 weeks post-op: WBAT

WBAT = weight bearing as tolerated, TTWB = toe touch weight bearing.

### Case 1

**Figure 2:**
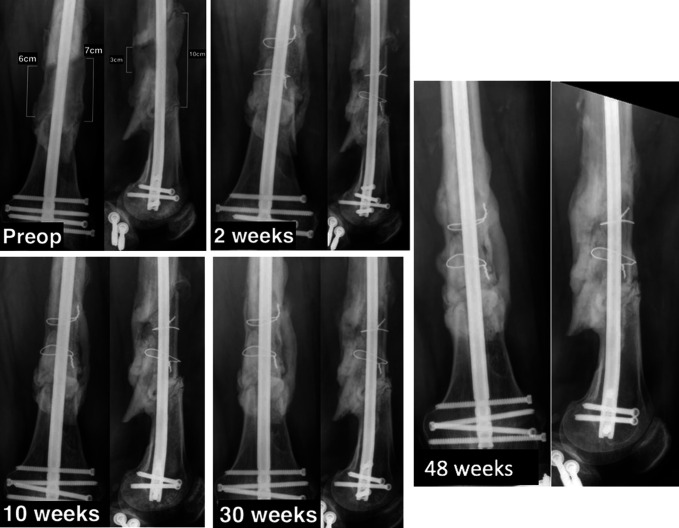
Case 1. Preoperative, 2 weeks, 10 weeks, 30 weeks, and 48 weeks follow-up radiographs.

This is a 38-year-old man who had a segmental left femur fracture with 10 cm bone loss. Six months before presentation to our office, the patient had sustained an open, segmental left femur fracture after being hit by a mass transit vehicle. Initially, he underwent irrigation and débridement and was placed in an external fixator. Two days later, stage one of the induced membrane technique was performed along with intramedullary nail fixation. He was then referred to one of the authors 6 months after his stage one procedure and subsequently underwent spacer removal, allograft strut placement, and bone grafting using Reamer Irrigator Aspirator (RIA) (DePuy Synthes) autograft from his contralateral femur. After the second stage surgery, as described previously, the patient was allowed to bear weight on left lower extremity for transfers and was made toe-touch weight bearing otherwise. One month after surgery, the patient's weight bearing was advanced to 50% of body weight. Eight weeks after surgery, he was allowed to bear weight as tolerated. He had no complications and at his last clinic visit was ambulating without pain and without assistive devices. His knee range of motion was 0° to 115° and had returned to competitive rally car driving. Preoperative radiographs and radiographs at 2, 5, 10, and 28 weeks postoperatively are presented in Figure 2. The last clinical follow-up was obtained at 11 months postoperatively.

### Case 2

This is a 40-year-old woman who sustained an open femur fracture with notable bone loss after being hit by a mass transit vehicle. She initially underwent irrigation and débridement with the placement of external fixation. She underwent two more débridements, and at the time of her second debridement, her soft-tissue envelope was appropriate for stage 1 of the induced membrane technique. A retrograde nail was passed in a standard fashion to maintain length and rotational stability of the femur with a PMMA spacer.

**Figure 3:**
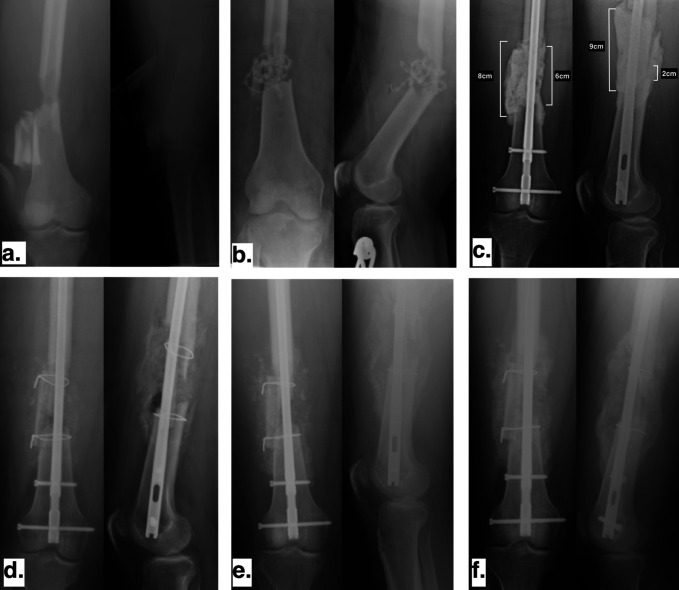
Case 2. (**A**) Injury radiograph. (**B**) Radiograph showing initial irrigation and debridement with the placement of antibiotic beads. (**C**) Radiograpg showing stage 1 of the induced membrane technique and fixation with intramedullary nail. (**D**) Radiograpg showing stage 2 of the induced membrane technique with placement of fibular allograft strut posterolaterally. (**E**) Radiographs at the two-week follow-up. (**F**) Radiographs at the 6-month follow-up.

Six weeks after stage 1, the patient returned to the operating room for spacer removal and bone grafting. The void was then packed with autograft bone harvested from the PSIS and a fibular strut was placed. The patient was made immediately toe-touch weight bearing. At 6 weeks, the patient required a return to the operating room for a knee contracture. She was weight bearing as tolerated by 3 months and by 6 months was able to ambulate without pain, had knee range of motion from 0° to 100°, and returned to work. Preoperative radiographs, 2 weeks post-op, and 6 months follow-up radiographs are shown in Figure 3. The last clinical follow-up was obtained at 6 months postoperatively. The patient did not return for other scheduled visits.

### Case 3

**Figure 4:**
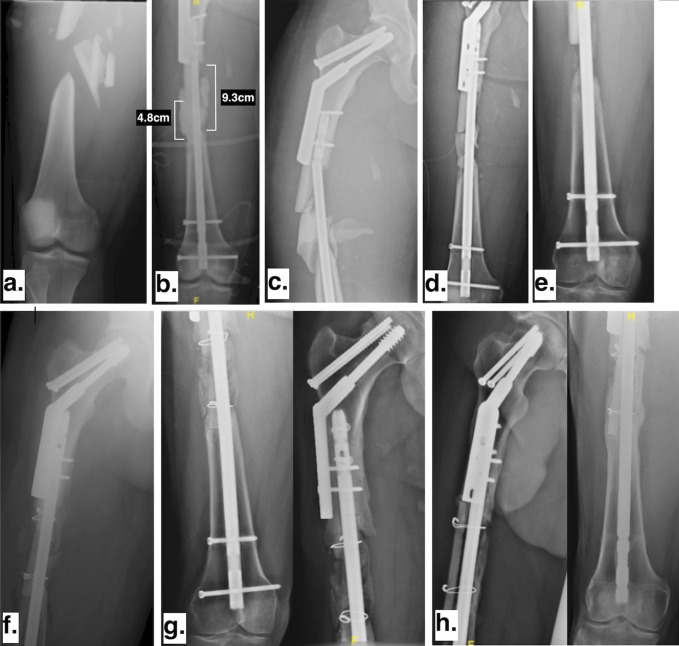
Case 3. (**A**) Injury radiograph. (**B**) Radiograpg showing initial fixation with intramedullary nail and cement spacer, proximally distal part of sliding hip screw can be seen. (**C**) Radiograpg showing failure of intramedullary nail at the distal end of sliding hip screw. (**D**) Radiograpg showing revision intramedullary nail and placement of new cement spacer. (**E**) Radiograpg showing second stage of induced membrane technique with placement of iliac crest autograft. (**F**) Radiograpg showing revision of nonunion with repeat second stage of induced membrane technique, this time with addition of fibular strut allograft. (**G**) Radiograpg showing six months after placement of fibular strut allograft. (**H**) Radiograpg showing fifteen months after placement of fibular strut allograft.

This is a 30-year-old man who sustained an open, segmental right femoral shaft fracture and an ipsilateral femoral neck fracture after being hit by a vehicle while riding a scooter. He initially underwent irrigation and débridement of the open fracture, retrograde intramedullary nail fixation of the right femur, and dynamic hip screw fixation of the ipsilateral femoral neck (Figure 4). The patient was instructed to be nonweight bearing on the right leg postoperatively; however, 3 weeks later, he sustained a fall, which resulted in failure of the intramedullary nail at the level of distal extent of the dynamic hip screw construct. The patient then underwent revision of the intramedullary fixation and stage 1 of the induced membrane technique after the segment of devitalized diaphysis was removed. The segmental defect of the femur diaphysis measured 9.3 cm medially and 4.8 cm laterally. During this period, the patient remained nonweight bearing on the right leg. One month after stage 1, the patient underwent stage 2 with the removal of the antibiotic spacer and placement of autograft bone from the iliac crest and proximal tibia. The patient was made nonweight bearing on the right leg for 12 weeks, advanced to toe-touch weight bearing for three more weeks, and finally progressive weight bearing. However, the patient continued to have pain at the fracture site. Nine months later, he was diagnosed with a right femur nonunion. He was referred to the author and underwent a repeat autograft bone grafting of the right femur defect with RIA (Depuy Synthes). At that time, placement of a fibular strut allograft was performed as described above. The patient was made toe-touch weight bearing after surgery and advanced to partial weight bearing of 25% to 50% body weight 6 weeks after. Ten weeks after surgery, he was advanced to partial weight bearing of 50% to 75% body weight. The patient was finally advanced to full weight bearing 5 months after the last surgery. At the last visit, the patient was fully weight bearing without pain and had knee range of motion from 0° to 100° (Figure 4). The last clinical follow-up was obtained at 15 months postoperatively.

## Discussion

Segmental bone defects remain a challenging complication in limb salvage. Contemporary techniques include induced membrane techniques, distraction osteogenesis, primary shortening, vascularized fibular graft transfer, and amputation.^27,28^ Treatment must be tailored to each patient's circumstance and clinical presentation.^27,28^ The Masquelet original induced membrane technique remains one of the mainstays of the limb salvage bony reconstruction ladder.^1^ This technique has been expanded to include reconstruction efforts of the long bones of the upper extremity, pediatric reconstruction, and even hand reconstruction.^5,6,7,8^

Studies evaluating massive intercalary allograft reconstruction after tumor resection have shown that the allograft usually unites to the host bone and provides structural support to the limb; however, high rates of mechanical failures can complicate these intercalary reconstructions.^29,30^ In contrast to these studies, where an intercalary allograft that matched the size of the defect was used, using a fibular strut allograft involves only a fraction of the circumferential area and serves as an adjunct to the Masquelet technique, providing structural support while host autograft is incorporating. This theoretically reduces the rate of mechanical failure seen with intercalary allografts.

Repair and healing of mechanically stable bone allograft constructs occurs at cortical to cortical or medullary to medullary junctions.^31-33^ At the cortical-cortical junction, healing takes place by bridging external callus that originates from the periosteum of the host bone.^33^ At the medullary to medullary junction, healing takes place because fibrovascular repair tissue from the host bone invades the marrow spaces of the allograft and deposits seams of reparative bone on the surfaces of the trabeculae of the allograft, uniting it to the trabeculae of the host bone.^33^ Enneking and Mindell^31^ showed that although the inner zone of the allograft remained necrotic and essentially acellular, the structure of the Haversian systems remained intact even after 5 years in vivo.

El-Alfy et al^34^ presented the result of 15 patients with segmental skeletal defects who were treated with the induced membrane technique using a free nonvascularized fibular autograft. They placed the fibula strut autograft into the medulla of the bone proximal and distal to the defect and, if needed, stabilized it with screws. Thirteen of the 15 patients achieved complete union, and two required regrafting because of nonunion. One patient had a deep wound infection. In contrast to their study, we used fibular strut allograft that eliminates fibular donor site morbidity and autograft harvest from RIA or the PSIS. This combination allows for increased structural stability and large volume autograft harvest with limited donor site morbidity.

Contemporary use of the induced membrane technique often involves stabilization of the fracture with an intramedullary nail or a plate, with the nail having the advantage of being load sharing.^28^ The amount of load endured by the nail is proportional to the stability of the fracture. When locking screws are used, physiologic loads are transmitted to the proximal and distal ends of the nail through the interlocking screws, which results in four-point bending stress on the screws.^35^ Therefore, intramedullary nails fail in predictable patterns. Locked nails fail by screw breakage or fracturing of the nail at the locking hole sites, most commonly at the proximal hole of the distal interlocking screw.^36^ In a study of immediate weight bearing after intramedullary nailing of femoral fractures, Brumback et al^37^ showed that stability of the fixation is dependent on the diameter of the interlocking screw in relationship to the nail diameter. Therefore, screws with the largest diameter possible should be used. Intramedullary reaming can also be used to increase the contact area between the nail and the cortical bone, and it can also allow insertion of a larger diameter nail, which improves the strength of the fixation. Biomechanically, reamed nails provide equal or better fixation than unreamed nails.^38,39^ By definition, segmental femur fractures will have poor or no cortical contact and the intramedullary device will bear all of the load if early weight bearing is allowed. In these segmental fractures, or fractures with substantial bone loss, the risk of implant failure is present and is one of the most challenging complications of these fractures.^36,40^ The time to full weight bearing when reconstructing segmental femur fractures depends on the fixation strategy used. Masquelet and Begue^3^ described a series of 35 patients with segmental defects who underwent the induced membrane technique reconstruction. For the 29 patients with lower extremity defects, full weight bearing was achieved at a mean time of 8.5 months.

Chapman^41^ described a surgical technique for the treatment of open femur fractures with considerable bone loss. The initial injury was treated with irrigation and débridement and delayed primary closure. After the wound had healed (usually approximately three weeks), a closed intramedullary bone-grafting and nailing procedure was performed. Autograft bone obtained from the reamings, and the greater trochanter was introduced into the bone defect using a plastic thoracostomy tube introduced through the reamed femoral canal. Two of the three patients went on to union. Postoperatively, the patients were placed in balanced traction until callus could be seen bridging the defect, which in the two patients with successful results was seen at 6 weeks.

Our technique describes the use of a fibular strut allograft to aid in bone regeneration and structural stability during stage two of the Masquelet induced membrane method. The fibular strut allograft allows for a structural scaffold, which in combination with intramedullary fixation allows for earlier weight bearing. Wires are used to encircle the strut and keep it fixed to the proximal and distal fracture fragments. The earlier weight bearing also allows for more vigorous rehabilitation and may speed functional recovery. In addition, this technique has proved useful in treating patients with delayed time to referral for stage II of the Masquelet technique, as evidenced by two of the patients in this case series.

As with all open fractures, early antibiotics and meticulous débridement are key to optimizing outcomes. Reestablishing length, rotation, and alignment are also vital in the final fixation of long bones, and close attention should be given to these factors. Even earlier weight bearing may be considered. Furthermore, a similar technique way be useful when plating is used for fracture fixation.

Our study has some limitations. We had a small number of cases and relatively short follow-up. The amount of mechanical contribution added by the fibular allograft is unknown. Finally, introducing large segments of allograft bone could increase the risk of infection,^30^ particularly in the setting of a previous open fracture site. Although the fibular strut allograft is small compared with the segmental area that is filled with autograft bone, it is a risk that cannot be obviated.

## Conclusion

Using a fibular strut allograft is a useful adjunct to the induced membrane technique in the treatment of femur fractures with segmental bone loss. It provides mechanical support while the host bone heals, which prevents implant failure while allowing for early weight bearing and functional recovery.
